# Trends in Pneumoconiosis Deaths — United States, 1999–2018

**DOI:** 10.15585/mmwr.mm6923a1

**Published:** 2020-06-12

**Authors:** Jessica L. Bell, Jacek M. Mazurek

**Affiliations:** ^1^Association of Schools and Programs of Public Health/CDC Public Health Fellowship Program; ^2^Respiratory Health Division, National Institute for Occupational Safety and Health, CDC

Pneumoconioses are preventable occupational lung diseases caused by inhaling dust particles such as coal dust or different types of mineral dusts (*1*). To assess recent trends in deaths associated with pneumoconiosis, CDC analyzed multiple cause-of-death data*^,^^†^ for decedents aged ≥15 years for the years 1999–2018, and industry and occupation data collected from 26 states^§^ for the years 1999, 2003, 2004, and 2007–2013. During 1999–2018, pneumoconiosis deaths decreased by 40.4%, with the exception of pneumoconiosis attributed to other inorganic dusts (e.g., aluminum, bauxite, beryllium, iron, and tin oxide), which increased significantly (p-value for time trend <0.05). The largest observed decreases in pneumoconiosis deaths were for those associated with coal workers’ pneumoconiosis (69.6%) and silicosis (53.0%). Asbestosis was the most frequently reported pneumoconiosis and was associated with working in the construction industry. The ongoing occurrence of deaths associated with pneumoconiosis underscores the importance of occupational dust exposure reduction, early case detection, and continued surveillance to monitor trends.

The CDC National Vital Statistics System’s multiple cause-of-death data for 1999–2018 were analyzed for decedents aged ≥15 years. For this analysis, decedents were identified using death certificates listing pneumoconiosis as the underlying^¶^ or contributing cause of death and included deaths with the following *International Classification of Diseases, Tenth Revision* (ICD-10) codes: J60 (coal workers’ pneumoconiosis), J61 (pneumoconiosis due to asbestos and other mineral fibers, [asbestosis]), J62 (pneumoconiosis due to dust containing silica, [silicosis]), J63 (pneumoconiosis due to other inorganic dust [applies to berylliosis, a disease caused by exposure to beryllium; pulmonary siderosis, a disease most common in workers exposed to metal fumes during welding; and other diseases]), J64 (unspecified pneumoconiosis), J65 (pneumoconiosis associated with tuberculosis), and J66 (airway disease due to specific organic dust [applies to byssinosis, a disease caused by prolonged inhalation of textile fiber dust]). Death rates per 1 million population were age-adjusted by applying age-specific death rates to the 2000 U.S. Census standard population.** Industry and occupation data were available from 26 states for 1999, 2003, 2004, and 2007–2013 and coded^††^ in accordance with the U.S. Census 2000 Industry and Occupation Classification System.^§§^ Cause-of-death data from the 26 states were compiled using CDC’s National Occupational Respiratory Mortality Surveillance system.^¶¶^ Data were processed using SAS software (version 9.4; SAS Institute), and Joinpoint regression software (version 4.8.0.1; National Cancer Institute) was used to analyze time trends in deaths and log transformed death rates.

During 1999–2018, a total of 43,366 decedents aged ≥15 years had pneumoconiosis listed on their death certificates, including 17,578 (40.5%) for whom pneumoconiosis was the underlying cause of death. Among all pneumoconiosis decedents, 17,797 (41.0%) were aged 75–84 years, and nearly all were male (41,777; 96.3%), white (41,029; 94.6%), and non-Hispanic (42,339; 97.6%). Asbestosis was associated with approximately three fifths of the deaths (26,059; 60.1%), followed by coal workers’ pneumoconiosis (11,203; 25.8%), and unspecified pneumoconiosis (3,409; 7.9%) (Table 1).

**TABLE 1:** Pneumoconiosis mortality time trends among decedents aged ≥15 years, by disease* and year — United States, 1999–2018

Year	No. of deaths (rate)^†^							
	Total	Coal workers’ pneumoconiosis	Asbestosis	Silicosis	Pneumoconiosis attributed to other inorganic dusts	Unspecified pneumoconiosis	Pneumoconiosis associated with tuberculosis	Airway disease attributed to specific organic dust
1999	**2,738 (12.8)**	1,002 (4.7)	1,258 (5.8)	185 (0.9)	12 (—)^§^	284 (1.3)	5 (—)	7 (—)
2000	**2,859 (13.2)**	949 (4.4)	1,486 (6.8)	151 (0.7)	10 (—)	263 (1.2)	7 (—)	10 (—)
2001	**2,743 (12.4)**	886 (4.0)	1,449 (6.6)	163 (0.7)	10 (—)	233 (1.1)	7 (—)	10 (—)
2002	**2,715 (12.2)**	858 (3.8)	1,467 (6.6)	146 (0.6)	22 (0.1)	226 (1.0)	6 (—)	9 (—)
2003	**2,635 (11.6)**	772 (3.4)	1,464 (6.5)	177 (0.8)	12 (—)	210 (0.9)	6 (—)	8 (—)
2004	**2,524 (11.0)**	703 (3.1)	1,460 (6.4)	165 (0.7)	16 (—)	185 (0.8)	5 (—)	8 (—)
2005	**2,425^¶^ (10.4)**	652 (2.8)	1,416 (6.1)	160 (0.7)	19 (—)	189 (0.8)	7 (—)	7 (—)
2006	**2,308 (9.7)**	654 (2.8)	1,340 (5.7)	126 (0.5)	23 (0.1)	176 (0.7)	0 (—)	7 (—)
2007	**2,189 (9.1)**	524 (2.2)	1,393 (5.8)	122 (0.5)	9 (—)	144 (0.6)	5 (—)	5 (—)
2008	**2,155 (8.8)**	470 (1.9)	1,341 (5.5)	146 (0.6)	18 (—)	191 (0.8)	4 (—)	2 (—)
2009	**1,993 (8.0)**	480 (1.9)	1,255 (5.1)	121 (0.5)	15 (—)	140 (0.5)	2 (—)	1 (—)
2010	**2,028 (8.0)**	486 (1.9)	1,308 (5.2)	101 (0.4)	12 (—)	131 (0.5)	2 (—)	1 (—)
2011	**1,890 (7.2)**	409 (1.6)	1,243 (4.8)	88 (0.3)	17 (—)	140 (0.5)	4 (—)	5 (—)
2012	**1,850 (6.8)**	399 (1.4)	1,208 (4.5)	103 (0.4)	14 (—)	136 (0.5)	1 (—)	2 (—)
2013	**1,859 (6.8)**	361 (1.3)	1,229 (4.5)	111 (0.4)	22 (0.1)	145 (0.5)	2 (—)	1 (—)
2014	**1,790 (6.4)**	363 (1.3)	1,218 (4.4)	84 (0.3)	17 (—)	115 (0.4)	0 (—)	2 (—)
2015	**1,735 (6.0)**	323 (1.1)	1,188 (4.1)	105 (0.4)	25 (0.1)	107 (0.4)	2 (—)	2 (—)
2016	**1,662 (5.6)**	300 (1.0)	1,142 (3.9)	73 (0.2)	16 (—)	140 (0.4)	2 (—)	3 (—)
2017	**1,636 (5.4)**	307 (1.0)	1,102 (3.7)	98 (0.3)	17 (—)	118 (0.4)	1 (—)	5 (—)
2018	**1,632 (5.3)**	305 (1.0)	1,092 (3.5)	87 (0.3)	25 (0.1)	136 (0.4)	2 (—)	2 (—)
**Total**	43,366** (8.6)	**11,203 (2.2)**	**26,059 (5.2)**	**2,512 (0.5)**	**331 (0.1)**	**3,409 (0.7)**	**70 (0.0)**	**95 (0.0)**
Time trends								
Slope^††^	1999–2002 = −19.96	1999–2008 = −58.29^§§^	1999–2001 = 102.49^§§^	1999–2018 = −5.04^§§^	1999–2018 = 0.43^§§^	1999–2007 = −15.13^§§^	1999–2018 = −0.18^§§^	1999–2009 = −0.96^§§^
	2002–2009 = −102.51^§§^	2008–2018 = −20.63^§§^	2001–2018 = −23.90^§§^			2007–2018 = −3.09^§§^		2009–2018 = 0.13
	2009–2018 = −45.83^§§^							
APC^¶¶^	1999–2001 = −0.88	1999–2018 = −8.56^§§^	1999–2002 = 4.02	N/A***	N/A***	N/A***	N/A***	N/A***
	2002–2018 = −5.22^§§^		2001–2018 = −3.94^§§^					

**Source:** CDC WONDER multiple cause-of-death data. https://wonder.cdc.gov/mcd.html.

**Abbreviations:** APC = annual percent change; N/A = not available.

* *International Classification of Diseases, Tenth Revision* codes: J60 (coal workers’ pneumoconiosis), J61 (pneumoconiosis due to asbestos and other mineral fibers, [asbestosis]), J62 (pneumoconiosis due to dust containing silica, [silicosis]), J63 (pneumoconiosis due to other inorganic dusts]), J64 (unspecified pneumoconiosis), J65 (pneumoconiosis associated with tuberculosis), and J66 (airway diseases due to specific organic dust).

^†^ Death rates per 1 million population were age-adjusted by applying age-specific death rates to the 2000 U.S. Census standard population.

^§^ Dashes indicate unreliable death rates because there were fewer than 20 deaths per year.

^¶^ Data were compiled using CDC WONDER’s record axis methodology, which differs from Healthy People 2020’s entity axis methodology. Healthy People 2020’s baseline total is 2,430. https://www.healthypeople.gov/node/5046/data_details.

** The sum of decedents is less than sum of disease-associated deaths because some decedents have more than one type of pneumoconiosis listed on their death certificate.

^††^ Calculated using death counts; the slope characterizes the direction of the disease trend (negative slope indicates decrease in deaths over time).

^§§^ p<0.05.

^¶¶^ Calculated using age-adjusted death rates.

*** APCs could not be calculated because of unreliable death rates or insufficient data to determine standard error.

During 1999–2018, the overall annual number of pneumoconiosis deaths decreased 40.4%; a significant decline began in 2002 (2,715 deaths) through 2018 (1,632) (p-value for time trend <0.05). Age-adjusted death rates (deaths per 1 million population) decreased from 12.8 in 1999 to 5.3 in 2018 (annual percent change = −0.88% during 1999–2001 and −5.22% during 2002–2018 [p-value for 2002–2018 time trend <0.05]).

Deaths decreased for all types of pneumoconiosis during the period studied, with the exception of those attributed to other inorganic dusts, which increased significantly from 12 deaths in 1999 to 25 in 2018 (108.3%; p<0.05). However, none of the distinct disease categories in this group increased significantly. The largest decreases over time were for deaths associated with coal workers’ pneumoconiosis (69.6%), from 1,002 in 1999 to 305 in 2018 (p-value for time trend <0.05), and silicosis (53.0%), from 185 in 1999 to 87 in 2018 (p-value for 2018 time trend <0.05]) (Table 1).

Age-adjusted death rates varied across geographic locations for each pneumoconiosis type (Table 2). The highest age-adjusted death rates for the 20-year period were in West Virginia for coal workers’ pneumoconiosis (59.8 per million population), Montana for asbestosis (20.0), Vermont for silicosis (2.3), and West Virginia for unspecified pneumoconiosis (24.1).

**TABLE 2:** Number of coal workers’ pneumoconiosis, asbestosis, silicosis, and unspecified pneumoconiosis-associated deaths* and age-adjusted death rates^†^ among persons aged ≥15 years, by state — United States, 1999–2018

State	No. of deaths (rate)^†^			
	Coal workers’ pneumoconiosis	Asbestosis	Silicosis	Unspecified
Alabama	120 (1.5)	818 (10.2)	41 (0.5)	51 (0.7)
Alaska	—^§^	39 (7.2)	—^§^	—^§^
Arizona	43 (0.4)	337 (3.2)	68 (0.6)	30 (0.3)
Arkansas	37 (0.7)	249 (4.8)	20 (0.4)	—^§^
California	155 (0.3)	1,844 (3.4)	105 (0.2)	48 (0.1)
Colorado	111 (1.6)	270 (4.1)	119 (1.8)	115 (1.7)
Connecticut	—^§^	327 (4.9)	13 (—)^¶^	—^§^
Delaware	—^§^	218 (14.2)	—^§^	—^§^
District of Columbia	—^§^	—^§^	—^§^	—^§^
Florida	184 (0.5)	1,667 (4.0)	68 (0.2)	49 (0.1)
Georgia	31 (0.3)	308 (2.5)	39 (0.3)	22 (0.2)
Hawaii	—^§^	56 (2.2)	—^§^	—^§^
Idaho	—^§^	177 (7.6)	27 (1.1)	11 (—)^¶^
Illinois	234 (1.1)	435 (2.1)	65 (0.3)	59 (0.3)
Indiana	133 (1.3)	216 (2.1)	53 (0.5)	35 (0.3)
Iowa	31 (0.5)	153 (2.6)	16 (—)^¶^	10 (—)^¶^
Kansas	12 (—)^¶^	134 (2.7)	11 (—)^¶^	—^§^
Kentucky	1,596 (22.1)	246 (3.5)	57 (0.8)	350 (4.9)
Louisiana	47 (0.7)	515 (7.4)	39 (0.5)	—^§^
Maine	—^§^	287 (10.8)	—^§^	—^§^
Maryland	34 (0.4)	728 (8.2)	26 (0.3)	23 (0.3)
Massachusetts	—^§^	641 (5.3)	19 (—)^¶^	—^§^
Michigan	79 (0.5)	687 (4.0)	80 (0.5)	35 (0.2)
Minnesota	13 (—)^¶^	502 (5.6)	59 (0.7)	—^§^
Mississippi	245 (5.3)	666 (14.0)	30 (0.6)	—^§^
Missouri	25 (0.2)	258 (2.5)	41 (0.4)	10 (—)^¶^
Montana	—^§^	363 (20.0)	19 (—)^¶^	—^§^
Nebraska	—^§^	102 (3.2)	—^§^	—^§^
Nevada	16 (—)^¶^	132 (3.7)	27 (0.7)	15 (—)^¶^
New Hampshire	—^§^	125 (5.6)	10 (—)^¶^	—^§^
New Jersey	34 (0.2)	1,318 (8.6)	40 (0.3)	30 (0.2)
New Mexico	75 (2.4)	96 (3.0)	51 (1.6)	113 (3.5)
New York	52 (0.2)	1,178 (3.5)	119 (0.4)	56 (0.2)
North Carolina	112 (0.7)	862 (5.8)	76 (0.5)	35 (0.2)
North Dakota	—^§^	56 (4.3)	—^§^	—^§^
Ohio	366 (1.8)	1045 (5.1)	204 (1.0)	139 (0.7)
Oklahoma	40 (0.7)	206 (3.3)	28 (0.4)	13 (—)^¶^
Oregon	—^§^	597 (8.8)	36 (0.5)	—^§^
Pennsylvania	3,258 (12.3)	1,553 (6.0)	268 (1.1)	636 (2.4)
Rhode Island	—^§^	122 (5.9)	14 (—)^¶^	—^§^
South Carolina	41 (0.5)	536 (7.2)	39 (0.5)	—^§^
South Dakota	—^§^	29 (1.8)	15 (—)^¶^	—^§^
Tennessee	273 (2.7)	515 (5.1)	52 (0.5)	59 (0.6)
Texas	107 (0.3)	2,106 (6.7)	157 (0.4)	52 (0.1)
Utah	89 (2.9)	112 (3.8)	45 (1.5)	63 (2.1)
Vermont	—^§^	61 (5.5)	27 (2.3)	—^§^
Virginia	1,300 (10.8)	894 (7.5)	44 (0.4)	326 (2.7)
Washington	19 (—)^¶^	1,322 (12.8)	36 (0.3)	12 (—)^¶^
West Virginia	2,191 (59.8)	516 (14.1)	58 (1.5)	887 (24.1)
Wisconsin	22 (0.2)	382 (3.8)	116 (1.2)	14 (—)^¶^
Wyoming	28 (3.3)	45 (5.3)	—^§^	35 (4.2)

**Source:** CDC WONDER multiple cause-of-death data. https://wonder.cdc.gov/mcd.html.

* Pneumoconiosis deaths attributed to other organic dusts or specific organic dust or associated with tuberculosis are not displayed because the numbers of cases were fewer than10 for each state.

**^†^** Death rates per 1 million population were age-adjusted by applying age-specific death rates to the 2000 U.S. Census standard population.

^§^ Suppressed because there were fewer than 10 decedents.

^¶^ Unreliable death rates because there were fewer than 20 deaths per state.

Industry and occupation data were available for 6,223 (96.7%) of 6,436 pneumoconiosis-associated deaths among persons aged ≥15 years from 26 states during 1999, 2003, 2004, and 2007–2013 (Table 3). Whereas the highest number of coal workers’ pneumoconiosis–associated deaths occurred among workers in the coal mining industry (1,331; 74.2%), and among mining machine operators (1,203; 65.0%), the highest number of asbestosis-associated deaths occurred among workers in the construction industry (820; 25.0%) and among pipe layers, plumbers, pipefitters, and steamfitters (264; 8.0%). The highest number of silicosis-associated deaths occurred among workers in the construction industry (63; 18.9%) and among mining machine operators (41; 12.3%).

**TABLE 3:** Top three industries and occupations associated with pneumoconiosis* deaths among persons aged ≥15 years, by disease^ꝉ^ — 26 states,^§^ 1999, 2003, 2004, and 2007–2013

Disease	Characteristic	No. (%)^¶^ of deaths
**Coal workers’ pneumoconiosis (n = 1,838)**		
**Industry**	Coal mining	1,331 (74.2)
	Construction	75 (4.1)
	Nonpaid worker	52 (2.8)
**Occupation**	Mining machine operators	1,203 (65.0)
	Laborers and freight, stock, and material movers	43 (2.3)
	Homemakers	41 (2.2)
**Asbestosis (n = 3,284)**		
**Industry**	Construction	820 (25.0)
	Industrial/Miscellaneous chemicals	162 (5.0)
	Not specified manufacturing industries	148 (4.5)
**Occupation**	Pipe layers, plumbers, pipefitters, and steamfitters	264 (8.0)
	Electricians	145 (4.4)
	Carpenters	110 (3.4)
**Silicosis (n = 333)**		
**Industry**	Construction	63 (18.9)
	Coal mining	25 (7.5)
	Foundries	19 (5.7)
**Occupation**	Mining machine operators	41 (12.3)
	Laborers and freight, stock, and material movers	21 (6.3)
	Construction laborers	14 (4.2)
**Unspecified pneumoconiosis (n = 792)**		
**Industry**	Coal mining	508 (64.1)
	Metal ore mining	34 (4.3)
	Construction	32 (4.0)
**Occupation**	Mining machine operators	485 (61.2)
	Laborers and freight, stock, and material movers	17 (2.1)
	Electricians	15 (1.9)

**Source:** National Institute for Occupational Safety and Health, CDC. https://webappa.cdc.gov/ords/norms-io2000.html.

* Excludes the following *International Classification of Diseases, Tenth Revision* codes because five or fewer deaths occurred in available industries or occupations: J63 (pneumoconiosis due to other inorganic dusts), J65 (pneumoconiosis associated with tuberculosis), and J66 (airway diseases due to specific organic dust).

^†^ *International Classification of Diseases, Tenth Revision* codes: J60 (coal workers’ pneumoconiosis), J61 (pneumoconiosis due to asbestos and other mineral fibers, [asbestosis]), J62 (pneumoconiosis due to dust containing silica, [silicosis]), J64 (unspecified pneumoconiosis), J65 (pneumoconiosis associated with tuberculosis), and J66 (airway diseases due to specific organic dust [including byssinosis]).

^§^ Colorado, Florida, Georgia, Hawaii, Idaho, Indiana, Kansas, Kentucky, Louisiana, Michigan, Nebraska, Nevada, New Hampshire, New Jersey, New Mexico, North Carolina, North Dakota, Ohio, Rhode Island, South Carolina, Texas, Utah, Vermont, Washington, West Virginia, and Wisconsin. States are where the death took place, not necessarily where the decedent had resided. Data were compiled using CDC’s National Occupational Respiratory Mortality Surveillance (NORMS) system. https://wonder.cdc.gov/wonder/help/mcd.html#Location.

^¶^ Percentage of total deaths associated with specific disease.

## Discussion

CDC previously examined pneumoconiosis mortality for 1968–2000 and reported decreases in death trends in all pneumoconioses with the exception of asbestosis, for which an increase was observed (*2*). In this report, the annual number of deaths associated with pneumoconiosis have continued to decline during 1999–2018 for all pneumoconioses with the exception of pneumoconiosis attributed to other inorganic dusts, which increased. In this category, berylliosis and siderosis were the most frequently reported diseases; however, there was no evidence of a change in death rates attributed to these conditions.

Each decade, the Healthy People Initiative develops new goals and objectives to improve the health of all Americans. The Healthy People 2020 Occupational Safety and Health Objective 4 set the goal of reducing pneumoconiosis deaths by 10% from the baseline of 2,430 deaths in 2005 to 2,187 deaths in 2020 (*3*). Results of this study indicate that the total number of pneumoconiosis deaths in 2018 was 1,632, a 32.8% decline from the baseline. If this trend continues, the goal will likely be surpassed in 2020.

The decline in overall pneumoconiosis mortality primarily reflects the decrease in coal workers’ pneumoconiosis and silicosis deaths, which together accounted for nearly one third (31.6%) of all pneumoconiosis-associated deaths reported during 1999–2018. The decline in coal workers’ pneumoconiosis–associated deaths likely reflects the reduction in the coal mining industry workforce (from 108,224 in 1999 to 98,505 in 2015)*** and legislative actions. For example, the 1969 Federal Coal Mine Health and Safety Act^†††^ required federal inspections of all coal mines, created enforceable safety measures, and added health protections and federal benefits for coal workers’ pneumoconiosis. Several other historical statutes^§§§^ have been enacted to improve miner safety and decrease disease mortality. Most recently, the 2014 final rule^¶¶¶^ of the Mine Safety and Health Administration (MSHA) standard on respirable coal mine dust lowered existing exposure limits from 2.0 mg of dust per cubic meter of air (mg/m^3^) to 1.5 mg/m^3^ at underground and surface coal mines, expanded medical monitoring for coal mine dust lung diseases, and made changes in dust monitoring systems to include the use of continuous personal dust monitors. Because of the long latency of coal workers’ pneumoconiosis, this new rule likely did not contribute to any decreases in mortality; however, adherence to this rule is expected to foster continued disease mortality reduction.

The decline in silicosis-associated deaths likely reflects the enactment of national compliance standards for silica dust exposure in 1971, implementation of disease prevention initiatives, and changes in industrial activity (*4*). The early standards, however, did not include measures such as medical surveillance requirements or employer and employee training about silica hazards. In 2016, the Occupational Safety and Health Administration (OSHA) published a final rule,**** for crystalline silica, lowering the permissible exposure limit to 50 *μ*g/m^3^ of air in all industries covered by the rule and included requirements to further protect employees (e.g., including exposure control, respiratory protection, hazard communication, medical surveillance, and recordkeeping). The rule also issued two separate standards, one for general industry and maritime and the other for construction, to tailor requirements to the respective industries’ hazards.

Asbestosis continues to be the most frequently reported cause of pneumoconiosis mortality, accounting for 60.1% of all pneumoconiosis deaths during 1999–2018. The number of annual asbestosis-associated deaths began to decline in 2001. This ongoing decrease likely reflects the cessation of asbestos mining, discontinued manufacturing of asbestos-containing products in the United States,^††††^ adoption of standards intended to control emissions of asbestos into the environment (*5*), and adoption of lower permissible exposure limits (*6*). In 1971, OSHA established a permissible exposure limit for asbestos at 12.0 fibers per cubic centimeter (f/cc) of air as an 8-hour time-weighted average. This initial permissible exposure limit was subsequently reduced to 5.0 f/cc in 1972, to 2.0 f/cc in 1976, to 0.2 f/cc in 1986, and to 0.1 f/cc in 1994.

Despite the decline in mortality and updated regulatory actions addressing occupational exposures to hazardous dusts, pneumoconiosis-associated deaths continue to occur, underscoring the need for maintaining exposure prevention measures and continued surveillance. Recent reports indicate the re-emergence of progressive massive fibrosis (the most severe form of coal workers’ pneumoconiosis) (*7*), new tasks and occupations (e.g., quartz countertop installation and hydraulic fracturing) that put workers at an increased risk for silicosis (*8*), continued importation of asbestos-containing materials for domestic consumption, and an increase in prevalence of other asbestos-associated diseases (e.g., malignant mesothelioma) (*9*). In addition, a 2019 significant new use rule^§§§§^for asbestos, promulgated to ensure that any discontinued uses of asbestos cannot re-enter the marketplace without Environmental Protection Agency review, still permits importation of asbestos into the United States; use of asbestos in gaskets, brakes, and chemical manufacturing; and asbestos mining.

The findings in this report are subject to at least five limitations. First, death records were not validated by medical records; therefore, results might be subject to misclassification. Second, some silicosis-associated deaths might not be work-related. For example, pneumoconiosis attributable to talc dust (ICD-10 code J62.0) in some decedents has been associated with use of illicit drugs (*10*); however, these pneumoconiosis-associated deaths were considered in this study to maintain comparability with previous studies and the Healthy People 2020 methods. Third, the industries and occupations represent the usual^¶¶¶¶^ industries and occupations entered on each death certificate, which might not be the industry and occupation in which the decedent’s exposure occurred. Fourth, the age-adjusted mortality rates might not correctly project disease frequency. The rates were calculated using data on the general population that might include those who are not at an occupational risk for developing the disease. Finally, because of small death counts, trends in pneumoconiosis attributable to other inorganic dusts could not be evaluated by distinct disease categories.

The decrease in pneumoconiosis-associated deaths during 1999–2018 indicates that prevention strategies are effective. The findings underscore the importance of maintaining primary prevention strategies to reduce exposures to respirable dusts, secondary prevention through early disease detection, and surveillance to monitor trends over time, in particular focusing on pneumoconiosis attributable to other inorganic dusts. Prevention strategies are available at the websites of OSHA (https://www.osha.gov/), MSHA (https://www.msha.gov/), and CDC’s National Institute for Occupational Safety and Health (https://www.cdc.gov/niosh/index.htm).

**Summary**

**What is already known about this topic?**

Pneumoconioses are a group of occupational lung diseases caused by inhaling organic dust and inorganic mineral dust particles. From 1968 to 2000, death rates for all pneumoconioses decreased with the exception of those for asbestosis. Although preventable, deaths continue to occur.

**What is added by this report?**

Pneumoconiosis deaths decreased from 2,738 deaths in 1999 to 1,632 in 2018, and age-adjusted death rates decreased from 12.8 to 5.3 per million population. All pneumoconioses decreased with the exception of pneumoconiosis attributed to other inorganic dusts.

**What are the implications for public health practice?**

Pneumoconiosis-associated deaths continue to occur, underscoring the importance of occupational dust exposure reduction, early case detection, and continued surveillance to monitor trends, with an increased focus on pneumoconiosis attributable to other inorganic dusts.

## Footnotes

* Each death record includes codes for up to 20 conditions derived from the “Cause of Death” section of the death certificate. https://www.cdc.gov/nchs/data/dvs/DEATH11-03final-acc.pdf.

† https://wonder.cdc.gov/wonder/help/mcd.html.

§ Colorado, Florida, Georgia, Hawaii, Idaho, Indiana, Kansas, Kentucky, Louisiana, Michigan, Nebraska, Nevada, New Hampshire, New Jersey, New Mexico, North Carolina, North Dakota, Ohio, Rhode Island, South Carolina, Texas, Utah, Vermont, Washington, West Virginia, and Wisconsin. States are where the death took place, not necessarily where the decedent had resided.

¶ Underlying cause of death is defined as “the disease or injury which initiated the chain of morbid events leading directly to death, or the circumstances of the accident or violence which produced the fatal injury.” https://wonder.cdc.gov/wonder/help/mcd.html#Source.

** https://wonder.cdc.gov/wonder/help/mcd.html#Age-AdjustedRates.

†† https://www.cdc.gov/niosh/topics/coding/.

§§
https://www.census.gov/topics/employment/industry-occupation/data/tables.2000.html .

¶¶ https://wonder.cdc.gov/wonder/help/mcd.html#Location.

***
https://www.msha.gov/sites/default/files/Data_Reports/DEC_15_2016_Historical_MIWQ_Employment_and_Production.pdf .

†††
https://www.msha.gov/45-years-federal-coal-mine-health-and-safety-act .

§§§
https://www.msha.gov/regulations/laws .

¶¶¶ https://www.govinfo.gov/content/pkg/FR-2014-05-01/pdf/2014-09084.pdf.

**** https://www.federalregister.gov/documents/2016/03/25/2016-04800/occupational-exposure-to-respirable-crystalline-silica.

†††† https://www.usgs.gov/centers/nmic/mineral-commodity-summaries.

§§§§ https://www.federalregister.gov/documents/2019/04/25/2019-08154/restrictions-on-discontinued-uses-of-asbestos-significant-new-use-rule.

¶¶¶¶
https://www.cdc.gov/nchs/data/misc/hb_occup.pdf .
